# An NF-κB–Dependent Role for JunB in the Induction of Proinflammatory Cytokines in LPS-Activated Bone Marrow–Derived Dendritic Cells

**DOI:** 10.1371/journal.pone.0009585

**Published:** 2010-03-08

**Authors:** Tiphanie Gomard, Henri-Alexandre Michaud, Denis Tempé, Kevin Thiolon, Mireia Pelegrin, Marc Piechaczyk

**Affiliations:** 1 Institut de Génétique Moléculaire de Montpellier, Centre National de la Recherche Scientifique, Montpellier, France; 2 Université Montpellier 2, Montpellier, France; 3 Université Montpellier 1, Montpellier, France; New York University, United States of America

## Abstract

**Background:**

Dendritic cells (DCs) play a key role in the induction of adaptive and memory immune responses. Upon encounter with pathogens, they undergo a complex maturation process and migrate toward lymphoid organs where they stimulate immune effector cells. This process is associated with dramatic transcriptome changes, pointing to a paramount role for transcription factors in DC activation and function. The regulation and the role of these transcription factors are however ill-defined and require characterization. Among those, AP-1 is a family of dimeric transcription complexes with an acknowledged role in the control of immunity. However, it has not been studied in detail in DCs yet.

**Methodology/Principal Findings:**

Here, we have investigated the regulation and function of one of its essential components, JunB, in primary bone marrow–derived DCs induced to maturate upon stimulation by *Escherichia coli* lipopolysaccharide (LPS). Our data show fast and transient NF-κB–dependent transcriptional induction of the *junb* gene correlating with the induction of the TNFα, IL-6, and IL-12 proinflammatory cytokines. Inhibition of JunB protein induction by RNA interference hampered the transcriptional activation of the TNF-α, IL-6, and IL-12p40 genes. Consistently, chromatin immunoprecipitation experiments showed LPS-inducible binding of JunB at AP-1–responsive sites found in promoter regions of these genes. Concomitant LPS-inducible NF-κB/p65 binding to these promoters was also observed.

**Conclusions/Significance:**

We identified a novel role for JunB—that is, induction of proinflammatory cytokines in LPS-activated primary DCs with NF-κB acting not only as an inducer of JunB, but also as its transcriptional partner.

## Introduction

Dendritic cells (DCs) are professional antigen-presenting cells playing a key role in the induction of adaptive and memory immune responses as well as in tolerance to self-antigens [1], [2]. In response to a variety of microbial and endogenous stimuli, they capture antigens from their environment, which is followed by a complex maturation process. For example, upon uptake of pathogens, DC maturation typically includes major changes in the repertoire of surface receptors, acquisition of a migratory phenotype towards lymphoid organs, secretion of soluble mediators such as pro-inflammatory cytokines like TNF-α, IL-6 or IL-12 and induction of costimulatory- and MHC class I and II molecules, which are essential for eventual stimulation of effector lymphocytes [1], [2].

To detect microbial products and certain non-microbial endogenous factors, DCs are equipped with different cell surface molecular platforms. These receptors are, not only instrumental for antigen uptake, but also for induction of DC maturation *via* the activation of various signaling pathways [1]. Among them, the family of evolutionary conserved Toll-like receptors (TLRs) is central to the regulation of protective immune responses in pathogen-infected hosts [3]. For example, TLR4 binds the lipopolysaccharide (LPS) from Gram-negative bacteria such as *E. coli* [3] and activates various intracellular signaling cascades. Yet, these pathways that, importantly here, include the NF-κB- and AP-1 transcription complex pathways, have essentially been studied in non-DC cells [4], [5], [6].

DC maturation is associated with marked transcriptome reprogramming. Upon infection by pathogens, more than 1000 mRNA level changes can be monitored in DNA array studies with both core responses common to all activators and pathogen-specific programs of gene expression (for a review, see ref. [7]). These studies point to a paramount role for transcription factors. Indeed, it is notable that in macrophages, which are phagocytes closely related to DCs, stimulation by *E. coli* LPS entails mRNA variations for at least 92 of the 1288 known transcription factors [8], indicating a high degree of complexity in the regulation of TLR4-induced genes. It is however important to bear in mind that, despite the lineage proximity, transcriptional programs show significant differences between macrophages and DCs [7]. This is, for example, illustrated by differential induction of co-stimulatory molecules upon TLR4 stimulation [9].

The ubiquitous AP-1 transcriptional complex comprises a large family of dimeric transcription factors binding to AP-1/TREs (TPA-Responsive Elements) or CREs (cAMP-Responsive Elements) DNA motifs found in many gene promoters and enhancers. This explains that AP-1 is involved in the control of many physiological functions. Among the best-studied AP-1 components are the Jun family proteins (c-Jun, JunB and JunD). They can either homodimerize or heterodimerize between them or heterodimerize with other transcription factors, the best known of which are the Fos family members (c-Fos, Fra-1, Fra-2 and FosB) [10], [11]. AP-1 activates or represses transcription, depending on the dimer composition, the target gene, the cell context, the extracellular environment and which intracellular signaling cascades are activated [10], [11]. Extensive investigations in many *in vitro* and *in vivo* experimental systems have shown that the individual AP-1 subunits have many independent functions [12], [13], [14]. An important consequence of this is that the role of each AP-1 protein must be studied individually in any given situation. Moreover, such studies are complicated by the fact that certain AP-1 proteins can oscillate between activator and repressor states according to a variety of circumstantial factors that include their post-transcriptional modifications. For example, c-Fos or c-Jun transactivating activities are stimulated by a number of specific phosphorylations (see ref. [15] and references therein), whereas the same proteins are turned into transcriptional repressors upon sumoylation [16], [17].

AP-1 has been implicated in the control of immune functions, which include cytokine gene induction, in non-DC contexts such as macrophages, B-, T- and mast cells where it can collaborate with NF-κB [14]. This collaboration relies on a variety of factors. Among those, one can cite frequent concomitant activations of AP-1 and NF-κB, the vicinity of many AP-1- and NF-κB-responsive DNA motifs in gene promoters and the possibility of physical interactions between the two transcriptional complexes (see [18], [19] for references). Additionally, NF-κB and AP-1 can cross-regulate their expressions in a variety of situations [18], [19]. Surprisingly, despite the well established role of AP-1 in the regulation of immunity, little is known on its implication in DC biology. Our present knowledge is essentially limited to studies in cultured DC cell lines with no real investigations in primary cells. Except for a few functional reports, these studies have characterized the expression of certain AP-1 components in DCs and detected variations in overall AP-1 activity upon stimulation of various sorts with no specific investigation on specific Jun or Fos proteins [7], [20], [21], [22], [23], [24] or on collaboration with other signaling pathways.

To begin to unravel AP-1 function in DCs, we have focused here on the role of its JunB component. As, to our knowledge, no DC cell line fully recapitulates all of the biological properties of primary DCs, we have conducted our study using primary mouse bone marrow-derived DCs (BMDCs) that were activated by *E. coli* LPS (referred to as “LPS” hereafter). Our choice of JunB is justified by several reasons. First, the *junb* gene is rapidly induced by a plethora of stimuli in many different cell types, including LPS-stimulated macrophagic and -pre-B lymphocytic cell lines [19], [25]. Transcriptome analyses have also allowed to identify *junb* mRNA changes in DCs [7]. Yet, no kinetic studies were conducted and no protein analysis was conducted. Second, JunB is known to regulate cytokine and chemokine genes in cell types other than DCs, although it is not always clear whether the mechanisms involved are direct or indirect [18], [19], [26], [27], [28], [29], [30], [31], [32], [33] (see discussion). Third, JunB is a direct transcriptional target of NF-κB in various situations that include stimulation of 70Z/3 pre-B cells by LPS [19]. Moreover, JunB functionally collaborates with NF-κB, a transcription factor with essential role in DC activation [34], to activate the CCR7 gene in this cell line. Using a combination of qPCR, chromatin-immunoprecipitation (ChIP) and RNA interference approaches, we show that transcriptional induction of endogenous JunB is under direct control of LPS-activated NF-κB and essential for subsequent activation of genes coding for the proinflammatory cytokines TNF-α, IL-6 and IL-12 in LPS-stimulated BMDCs. Our data also suggest a collaboration between NF-κB and JunB in the transcriptional induction of these genes.

## Materials and Methods

### BMDC Preparation and Cell Cultures

Bone-marrow-derived DCs were obtained from bone marrow precursors of 6 to 7-week-old 129/Sv/Ev mice as described in Winzler *et al.* [35]. Briefly, bone marrow cells were withdrawn from the femurs and tibias. Red cells were lysed using the ACK lysis buffer from Amersham. B- and T lymphocytes were eliminated by negative depletion using the anti-CD19 and anti-CD90 magnetic beads from Miltenyi Biotech and monocytes by adherence on plastic plates. Cells were cultured for 7 days in RPMI 1640 medium supplemented with 5% fetal calf serum, 2 mM Glutamine, 50 µM β-mercaptoethanol, 1 mM sodium pyruvate, 1 mM non essential amino acids, 200 U/ml GM-CSF and 200 U/ml IL-4 (Peprotech). The culture medium was replaced every 2 days. The purity of the BMDC population was assessed by flow cytometry after double MHC class II and CD11c labelling.

### BMDC Stimulation and IKK Pharmacological Inhibition

Contaminants in *E. coli* LPS preparations from various suppliers can activate TLR2. We therefore resorted to ultrapure *E. coli* LPS from Invivogen as it is tested for exclusive stimulation of TLR4. It was used at a concentration of 1 µg/mL. The BAY 11-7085 (Calbiochem) inhibitor was used at a final concentration of 10 µg/mL.

### qRT-PCR Analyses

Total RNA from BMDCs was prepared using the GenElute™ Mammalian Total RNA Miniprep kit from Sigma. After treatment with RNAse-free DNAse I (Promega), 1 µg of total RNA was used for cDNA synthesis using Oligo(dT)_15_ (Promega) and the Superscript III Reverse Transcriptase from Invitrogen according to the supplier specifications. After 10-fold dilution, 2 µl of cDNA were used for real-time PCR analysis using the Roche LightCycler 480 real-time PCR system. The sequences of the amplification primers are presented in **Table 1**. Data analyses were performed using the LightCycler software (Roche) and normalized with respect to invariant S26 mRNA levels.

**Table 1:** Oligonucleotide sequences for qRT-PCR (A) and ChIP (B) experiments.

**A**			
**JunB**	Forward	5′-CAGCTACTTTTCGGGTCAGG-3′	
	Reverse	5′-ACGTGGTTCATCTTGTGCAG-3′	
**c-Jun**	Forward	5′-CATAGCCAGAACACGCTTCC-3′	
	Reverse	5′-AGTTGCTGAGGTTGGCGTAG-3′	
**JunD**	Forward	5′-ATCGACATGGACACGCAAGG-3′	
	Reverse	5′-ACGTGGCTGAGGACTTTCTG-3′	
**IL-6**	Forward	5′-TTGCCTTCTTGGGACTGATGCT-3′	
	Reverse	5′-GTATCTCTCTGAAGGACTCTGG-3′	
**TNF-α**	Forward	5′-GTGACCTGGACTGTGGGCCTC-3′	
	Reverse	5′-GGCTCTGTGAGGAAGGCTGTG-3′	
**IL-12p40**	Forward	5′-CAGAAGCTAACCATCTCCTGGTTTG-3′	
	Reverse	5′-TCCGGAGTAATTTGGTGCTTCACAC-3′	
**IL-12p35**	Forward	5′-CACCCTTGCCCTCCTAAACC-3′	
	Reverse	5′-GGTTTGGTCCCGTGTGATGT-3′	
**S26**	Forward	5′-GAACATTGTAGAAGCCGCTGCTGTC-3′	
	Reverse	5′-AACCTTGCTATGGATGGCACAGCTC-3′	
**B**			
**JunB**	Enhancer	Forward	5′-TATCCCCTGAGTCCTGGCACC-3′
		Reverse	5′-CGCTGGCGTCACTGAGCTGAA-3′
**JunB**	Distal region	Forward	5′-CAGCCCCTTCAGAGAGTGGAG-3′
		Reverse	5′-GGCAGTGACACCATCAAGCCC-3′
**TNFα**	Promoter	Forward	5′-TCCTTGATGCCTGGGTGTCCC-3′
		Reverse	5′-GCAGACGGCCGCCTTTATAGC-3′
**TNFα**	Distal region	Forward	5′-CCACCCCACCCCTGCCATTTT-3′
		Reverse	5′-CTGGGTCACCTGGAACTCTCC-3′
**IL-6**	Promoter	Forward	5′-TCCAATCAGCCCCACCCACTC-3′
		Reverse	5′-GGTGGGCTCCAGAGCAGAATG-3′
**IL-6**	Distal region	Forward	5′-CCAAACATCCTCCCCCAAATC-3′
		Reverse	5′-GGCATCTCTCACTCACCATCT-3′
**IL-12p40**	Promoter	Forward	5′-AAGCACCAGGAGCAGCCAAGG-3′
		Reverse	5′-GCAGGGAGTTAGCGACAGGGA-3′
**IL-12p40**	Distal region	Forward	5′-CCCGATGCCCCTGGAGAAACA-3′
		Reverse	5′-GGAGCAGCAGATGTGAGTGGC-3′

### Immunoblotting Analyses

LPS-stimulated or untreated cells were scrapped from culture dishes and directly lysed in SDS-containing electrophoresis loading buffer. Total cell extracts were boiled for 5 minutes and fractionated by PAGE before electrotransfer on PVDF membranes (Millipore). Immunodetections were carried out as previously described [36], [37] using either a monoclonal antibody to JunB (kind gift from Dr M. Yaniv, Paris) or various antisera from SantaCruz Biotechnology directed to JunD (Sc-74), c-Jun (Sc-45), IκBα (Sc-371) and glyceraldehyde-3-phosphate dehydrogenase (GAPDH; Sc-25778). GAPDH was used as an internal invariant control in our experiments. Final detection was carried out using HRP-conjugated anti-antibody reagents from Santa-Cruz Biotechnology, the enhanced chemolumiscence (ECL) kit from Millipore and the Biomax XAR films from Kodak. When necessary chemoluminescence signals were quantified using the Genegnome device from Ozyme.

### Indirect Immunofluorescence Assay

Intracellular localisation of p65/RelA was followed up by indirect immunofluorescence on paraformaldehyde-fixed cells as described in Malnou et al. [38], [39] using the Sc-372 antibody from Santa-Cruz Biotechnology.

### TNFα, IL-6, IL-12p70 ELISA

TNFα, IL-6 and IL-12p70 were assayed from LPS-stimulated BMDCs culture supernatants using the ELISA Ready-SET-Go! kit from eBioscience.

### Flow Cytometry Analysis

Fluorescent monoclonal antibodies directed to CD11c, CMHII, CD40 and CD80 were obtained from BD Biosciences and Miltenyi Biotec. After cell staining, fluorescence was assayed using the FACSCalibur flow cytometer from BD Immunocytometry Systems and the data were processed using the Cellquest Pro and FlowJo softwares.

### SiRNA Transfections

For RNAi experiments, either a scrambled control or a smart-pool siRNAs (Dharmacon) against JunB (100 ng per 5×10^5^ cells) was transfected in BMDCs using Lipofectamine 2000 (Invitrogen) according to the manufacturer's recommendations.

### Chromatin Immunoprecipitation Analyses

10^6^ cells were fixed in 1% formaldehyde at room temperature for 7.5 min. Fixation was blocked with 125 mM glycine final. Cells were scraped and rinsed twice with phosphate-buffered saline (PBS), allowed to lyse in 1 ml of cell lysis buffer (5 mM PIPES pH 7.4, 85 mM KCl, 0.5% NP40+protease inhibitor cocktail from Boehringher Manheim) at 0°C for 10 min. Nuclei were recovered by centrifugation at 4°C and lysed in 500 µl nucleus lysis buffer (50 mM Tris-HCl pH 7.5, 1% SDS, 10 mM EDTA + protease inhibitor cocktail) at 0°C for 2 hours. 250 µl of each sample were sonicated (at maximal power) using the Bioruptor UCD-200 sonifier (Diagenode) through 30 cycles of 30 seconds ON and 30 seconds OFF. Samples were incubated under rotational agitation at 4°C overnight in the presence of 2 µg of either a specific antibody or a pre-immune serum taken as negative control. Antibodies (Sc73 and Sc-372 from Santa-Cruz Biotechnology for JunB and p65/RelA, respectively) were previously bound to Dynabeads (Invitrogen) according to the supplier's recommendations. Dynabeads-bound immunoprecipitates were sequentially washed once with a low salt buffer (50 mM Tris-HCl pH 7.5, 150 mM NaCl, 1% triton, 0.1% SDS, 1 mM EDTA), a high-salt buffer (50 mM Tris-HCl pH 7.5, 500 mM NaCl, 1% triton, 0.1% SDS, 1 mM EDTA) and a LiCl-containing buffer (20 mM Tris-HCl pH 7.5, 250 mM LiCl, 1% NP40, 1% Na deoxycholate, 1 mM EDTA) and, then, twice with a TE buffer (10 mM Tris-HCl pH 7.5, 1 mM EDTA). After elution in 250 µl of elution buffer (100 mM NaHCO_3_, 1% SDS), DNA-protein complexes were incubated at 65°C for 5 hours to reverse crosslinks. Proteins and contaminating RNA were digested by incubation in the presence of 100 µg/ml proteinase K and 100 µg/ml RNAse A at 45°C for 2 hours. DNA was purified on Nuclear Extract II columns (Macherey-Nagel) according to the manufacturer's recommendations and then subjected to qPCR analysis using the Roche LightCycler 480 real-time PCR system. The data were normalized with inputs taken from samples before the immunoprecipitation and treated under the same conditions. The primers used to amplify the relevant regions of the *junb* and cytokine gene promoters are listed in **Table 1**. In all cases, AP-1- and NF-κB site-devoid fragments located downstream of the genes of interest were used as negative controls to exclude non-specific binding of JunB or NF-κB (not shown).

## Results

### Induction of JunB in Dendritic Cells

**Figure 1:**
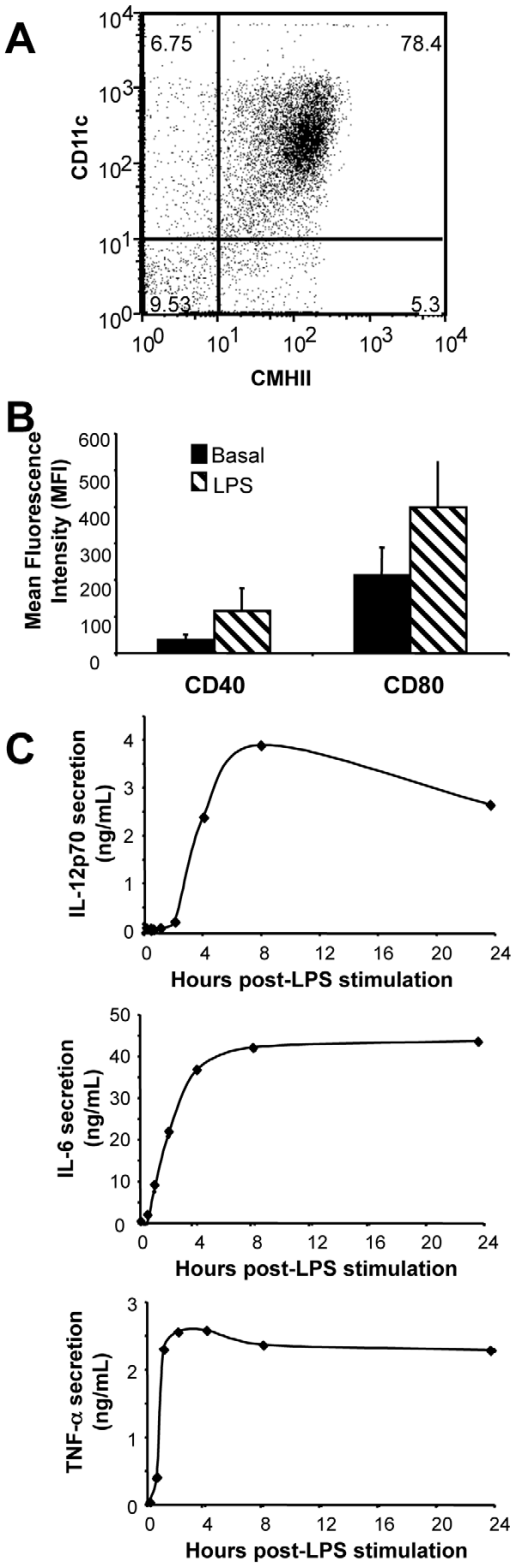
Characterization of *E. coli* LPS-induced BMDC maturation. (A) *Purity of BMDCs*. Bone marrow cells were cultured in the presence of GM-CSF and IL-4 for 7 days and analyzed by flow cytometry for the presence of CD11c and MHC II. (B) *Induction of CD40 and CD80 by LPS*. BMDCs were LPS-stimulated for 24 hours and analyzed for the induction of CD40 and CD80 by flow cytometry. Error bars correspond to standard deviation from 5 independent experiments. (C) *Cytokine induction*. BMDCs were LPS-stimulated for the indicated periods of time and cytokines were assayed from culture supernatant by ELISA. The presented data correspond to a representative experiment.

We exclusively used primary mouse bone marrow-derived DCs (BMDCs) in the herein study. They were classically amplified from bone marrow cells cultured for 7 days in the presence of GM-CSF+IL-4. Flow cytometry analysis of MHC class II and CD11c surface markers showed that they were reproducibly >80% pure (**Figure 1A**). Their proper activation by ultrapure *E. coli* LPS (see Materials and Methods) under our experimental conditions was verified by monitoring their morphological change by microscopic observation (not shown), the induction of the CD40 and CD80 cell surface markers by flow cytometry [1] (**Figure 1B**) and the induction of the proinflammatory cytokines TNF-α, IL-6 and IL-12 by ELISA (**Figure 1C**).

**Figure 2:**
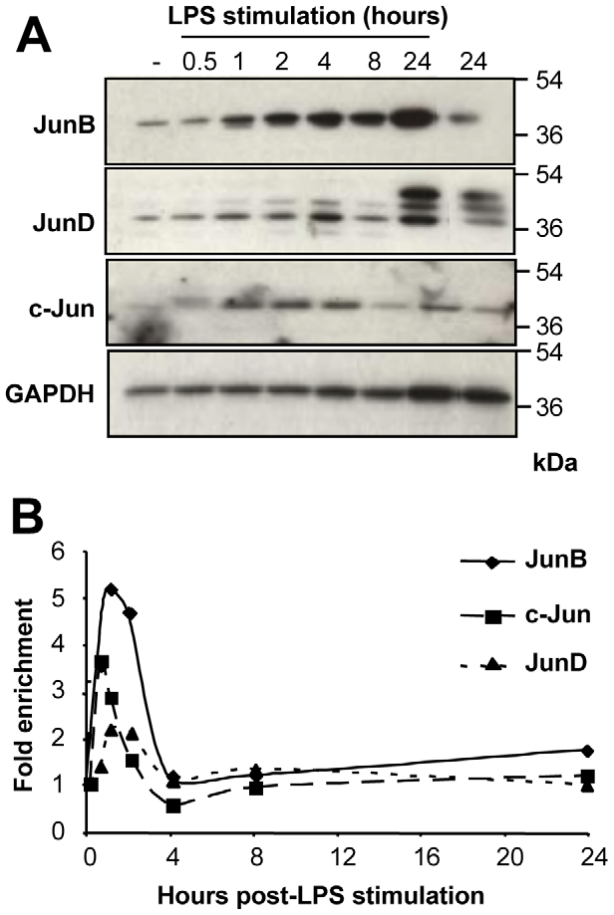
Expression of Jun proteins and *jun* mRNAs in LPS-stimulated BMDCs. (A) *Expression of the Jun proteins*. BMDCs were stimulated by LPS and c-Jun, JunB and JunD levels were assayed by immunoblotting. A representative experiment out of 5 is shown. GAPDH was used as an invariant electrophoresis loading control. JunD classically appeared as 3 bands whose exact molecular natures are still not elucidated. (B) *Expression of jun RNAs.* BMDCs were stimulated by LPS and *c-jun*, *junb* and *jund* mRNA levels were assayed by qRT-PCR. As inductions of *junb* and *c-jun* mRNAs peaked at different times ranging from. 5 to 1.5 hour post-stimulation, depending on the BMDC preparation, no error bar is presented. Instead, 1 representative experiment out of 5 is presented.

As a first step to our investigations, we asked whether JunB was induced by LPS treatment of BMDCs. To this aim, protein and mRNA levels were assayed by immunoblotting at various time points (**Figure 2A**) and qRT-PCR (**Figure 2B**), respectively. c-Jun and JunD being potential transcriptional partners (see discussion), we also followed them up in parallel. At the protein level, the 3 Jun proteins were induced, albeit to different degrees and with different kinetics. Our data can be summarized as follows: (i) the induction of JunB was fast (detectable as early as 1 hour post-stimulation) and continued steadily for the whole duration of the experiment, (ii) c-Jun was also induced rapidly, but more modestly than JunB, and this induction rapidly reached a plateau (by 1 hour post-stimulation) that was stable for the whole duration of the experiments and (iii) JunD was modestly induced during the initial phase of LPS stimulation but dramatically at the latest time point tested (24 hours). Strikingly, the patterns of mRNA accumulations were different from those of proteins with a transient induction by 1 hour post-stimulation followed by a rapid return to basal level. The extents of transient induction were however different among the 3 *jun* mRNAs: that of *jund* mRNA was the most modest (<2-fold), followed by that of *c-jun* (3-4-fold) and, then, that of *junb* (4-5-fold). Most probably due to, on the one hand, some variability between the various BMDC preparations we made and, on the other hand, the fact that primary DCs constitute a relatively heterogeneous population of cells at different stages of maturation, it must be noted that inductions peaked between 0.5 and 1.5 hours depending on the experiment with, however, reproducible return to basal level by 4 hours. c-Jun and JunB proteins being instable in other situations [37], [40], it is likely that their persistent accumulation in BMDCs was, at least partially, due to strong stabilization in these cell lineage. Supporting this possibility, cycloheximide chases indicated half-lives much longer than 4 hours for JunB in both LPS-stimulated and non-stimulated BMDCs (not shown). Moreover, increased mRNA translation contributing to higher accumulation in LPS-stimulated BMDCs cannot be excluded at this stage of investigation. Protein stabilization and/or increased mRNA translation should also account for the strong induction of JunD protein seen at 24 hours post-stimulation.

### NF-κB-Dependent Induction of the *junb* Gene in LPS-Stimulated BMDCs

**Figure 3:**
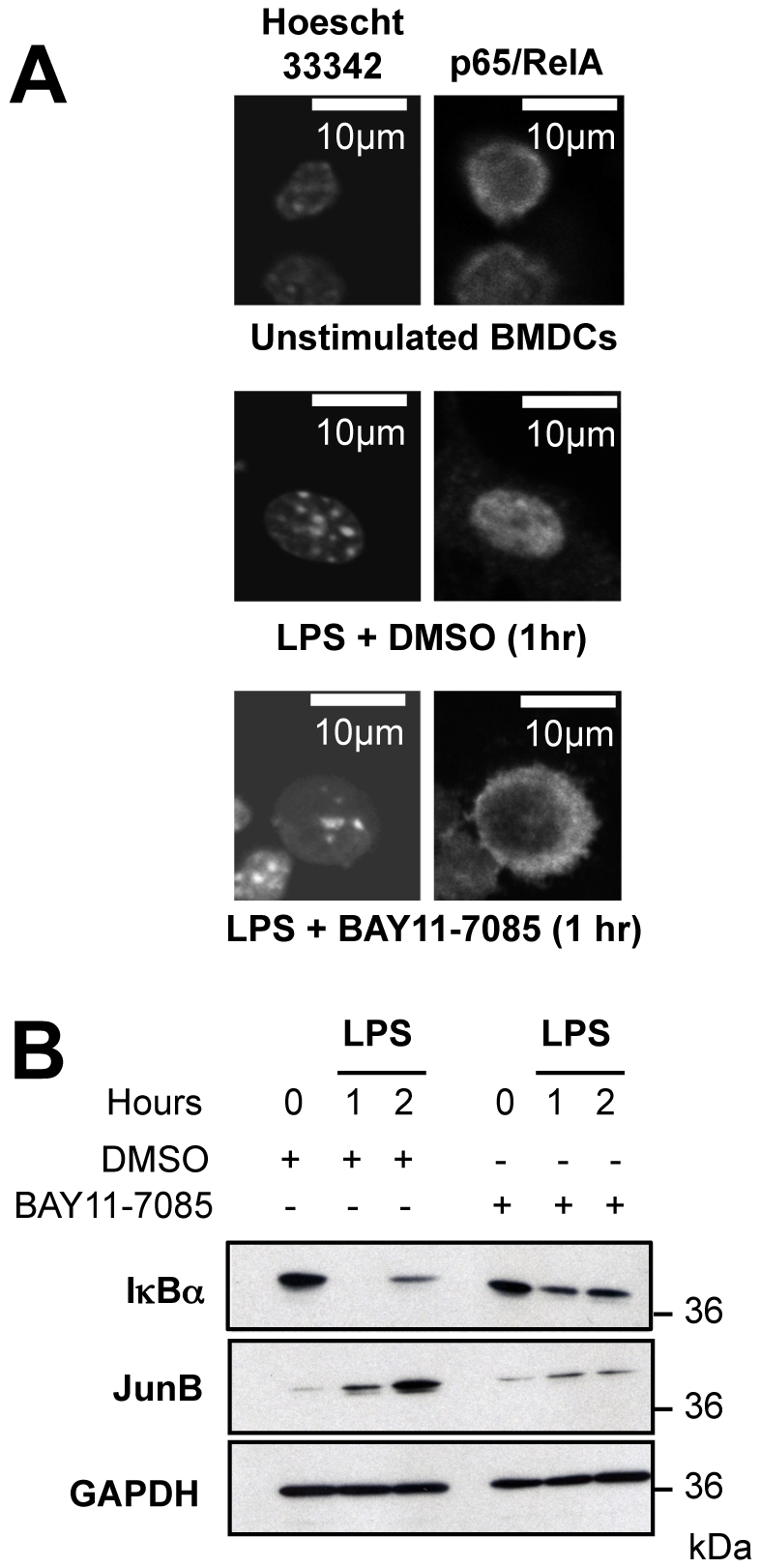
Involvement of the NF-κB pathway in JunB induction in BMDCs. (A) *Inhibition of p65/RelA nuclear translocation by BAY11-7085*. BMDCs were left unstimulated or were stimulated by LPS in the presence of either BAY 11-7085 or solvent (DMSO) for 1 hour. After cell fixation, nuclei were stained with Hoescht 33342 and p65/RelA was detected by indirect immunofluorescence. (B) *Inhibition of JunB induction by BAY11-7085*. BMDCs were stimulated by LPS in the presence of either BAY 11-7085 or solvent (DMSO) for various periods of time. The levels of IκBα and JunB were assayed by immunoblotting, taking GAPDH as an invariant electrophoresis loading control. The experiments were reproduced 3 times.

**Figure 4:**
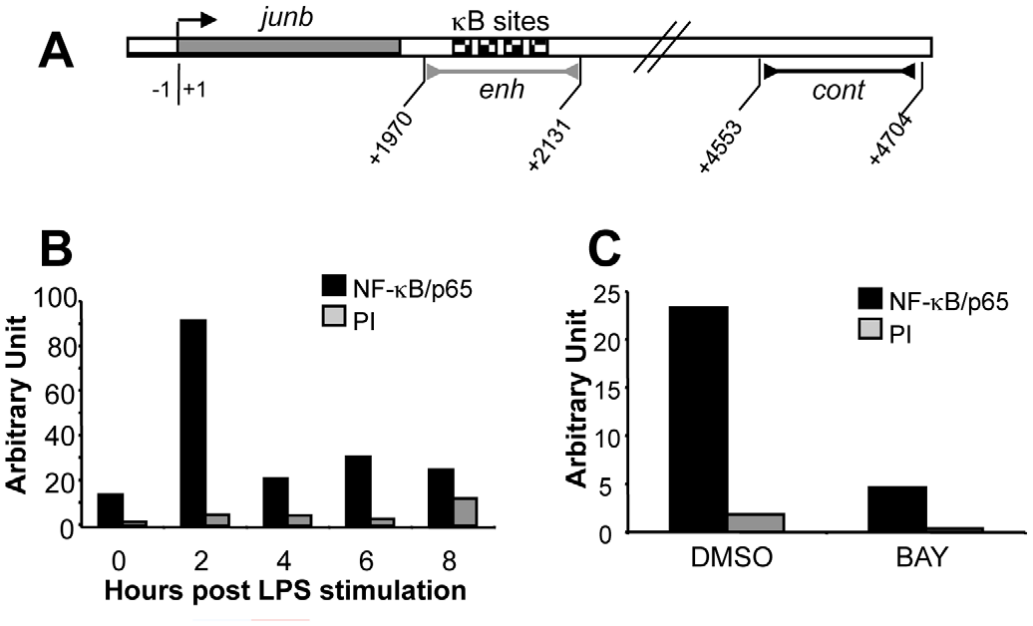
Direct transcriptional regulation of *junb* by NF-κB in LPS-stimulated BMDCs. (A) *Structure of the* junb *gene*. *junb* has no intron and is approximately 1900 bp long. The 4 kB sites are located within a 200 bp domain located approximately 200 bp downstream of the *junb* polyadenylation signal. The grey bar with the inverted arrows (enh) indicates the enhancer-containing fragment which is amplified in ChIP experiments to visualize NF-κB binding. The black bar with the inverted arrows (cont) located downstream the NF-kB enhancer region indicates the amplified negative control fragment used in the ChIP experiments to exclude non-specific NF-κB binding. The numbers indicate nucleotide position with respect to the transcription initiation site (+1). (B) *NF-κB binding in LPS-stimulated BMDCs*. BMDCs were stimulated for various periods of time with LPS before ChIP analysis. NF-κB binding in the enhancer region is presented in arbitrary units as well as the parallel negative control ChIPs carried out with a preimmune serum (PI). Calculations were made with respect to the amplification of the “cont” negative control fragment. The presented data are representative of 3 independent experiments (see text). (C) *Inhibition of NF-kB binding at the junb enhancer in the presence of BAY11-7085*. The same experiment as in B was conducted except that BAY11-7085 or DMSO was added together with LPS to BMDCs and incubation allowed to proceed for 1 hour.

Then, we investigated whether the induction of the *junb* gene was dependent on NF-κB in LPS-activated BMDCs. The p65/p50 NF-κB transcription complex is maintained inactive in the cytoplasm by the IκBα inhibitor but phosphorylation of IκBα by the IKK kinase upon activation of cells by various stimuli entails IκBα degradation within minutes and subsequent release of active NF-κB [1], [34]. Importantly, NF-κB induction is generally transient due to rapid resynthesis of IκBα in an NF-κB-dependent manner [1], [34]. To prevent NF-κB induction, siRNA against p65/RelA or transfection of a transdominant mutant of IκBα are classically used when carrying out experiments with established cell lines. We, however, could not resort to these approaches as anti-p65/RelA siRNAs revealed highly toxic to BMDCs (not shown) and unability to transfect BMDCs with plasmids is a major limitation of this system. Therefore, BMDCs were stimulated with LPS in the absence or in the presence of the BAY11-7085 inhibitor with well-established specificity for IKK [41], [42], [43] and JunB expression was analysed by immunoblotting. Two lines of evidence indicated efficient IKK inhibition in BMDCs by BAY-11-7085. First, p65/RelA, as assayed by indirect immunofluorescence, accumulated in the nucleus of 95% of LPS-stimulated cells in the absence of the drug but localized in the cytoplasm of 95% of LPS-stimulated cells in its presence (**Figure 3A**). Second, the degradation of IκBα was inhibited by BAY11-7085 (**Figure 3B** **)**. Interestingly, the data of **Figure 3B** showed a strong inhibition of JunB induction in BAY11-7085-treated BMDCs, which implicated, directly or indirectly, the NF-κB pathway. Worth of note, IκBα disappearance, which roughly corresponds to maximal NF-κB activity, was contemporary to *junb* mRNA induction. Additionally, we investigated the timing of NF-κB binding to the *junb* gene. In other cell types, transcriptional activation of *junb* by NF-κB has been reported to be principally mediated by an enhancer harboring 4 NF-κB-binding sites and located approximately 200 bp downstream the *junb* polyadenylation signal (**Figure 4A**; [31]). Consistently, chromatin immunoprecipation experiments (ChIP) showed a fast and transient inducible binding of NF-κB/p65 in this region that coincided with *junb* mRNA induction by LPS **(** **Figure 2B** **)**. A typical ChIP experiment is presented in **Figure 4B**. However, the peak of NF-κB fixation on the *junb* enhancer could vary between 1 and 2 hours from one experiment to the other due to the already mentionned heterogeneity of BMDCs. Moreover, in keeping with inhibition of both IκBα degradation and nuclear translocation of p65/RelA by BAY11-7085, binding of p65/RelA to the *junb* enhancer was dramatically reduced in the presence of the drug (**Figure 4C**). Thus, altogether our data indicate that the induction of JunB in LPS-stimulated BMDCs results from a direct transcriptional induction of the *junb* gene by NF-κB.

### Inductions of TNF-α-, IL-6- and IL-12p40 mRNAs Depend on That of JunB in LPS-Activated BMDCs

**Figure 5:**
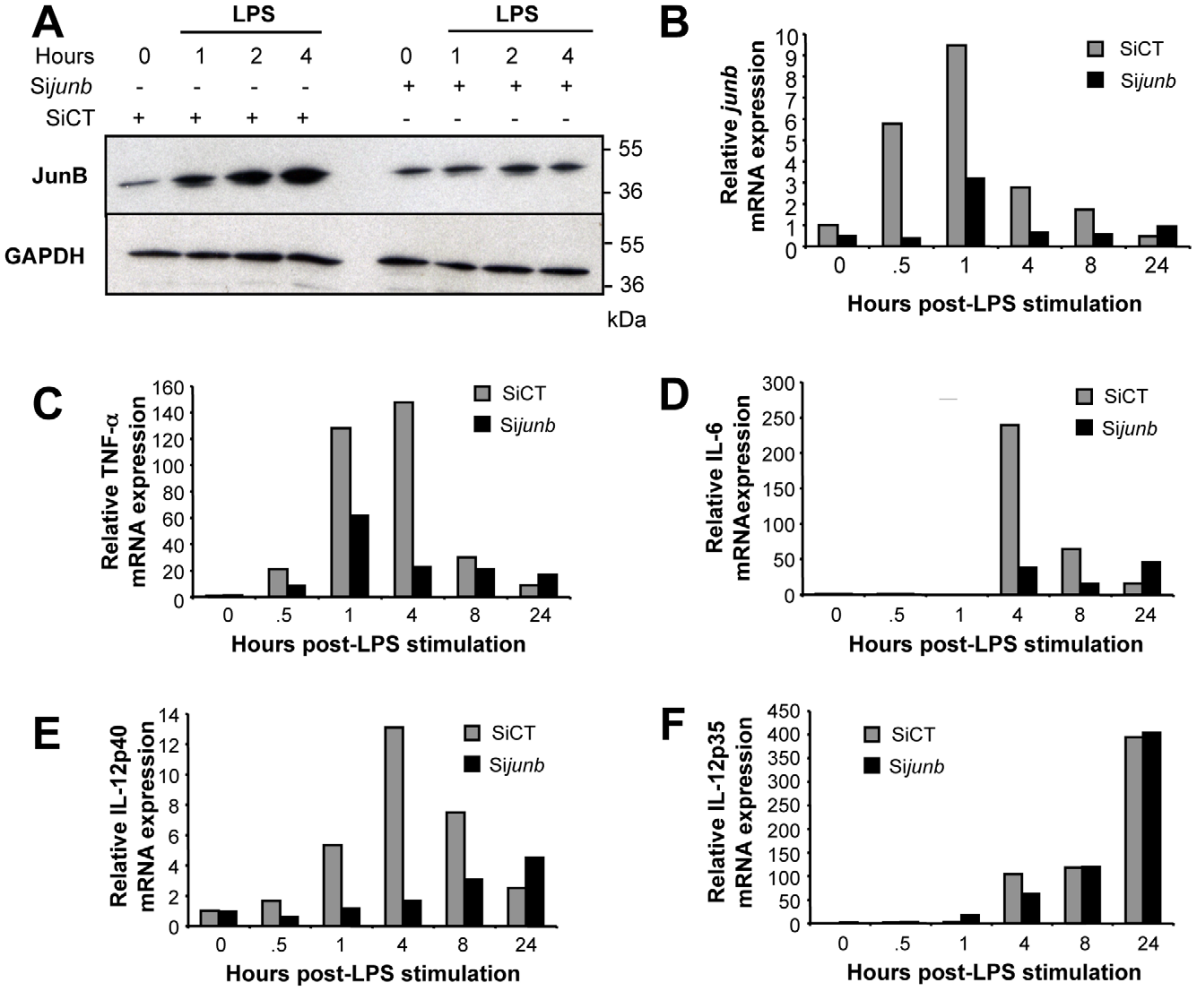
Involvement of JunB in the induction of TNFα, IL-6 and IL-12p40 mRNA induction in LPS-stimulated BMDCs. *Effect of the* junb *siRNA on JunB protein (A) and* junb *mRNA (B) inductions in LPS-stimulated BMDCs*. BMDCs were transfected with either a siRNA directed to *junb* mRNA (Si*junb*) or a control siRNA (SICT). 24 hours later they were stimulated, or not, by LPS and JunB protein and *junb* mRNA were assayed by immunoblotting and qRT-PCR, respectively. *(C, D, E and F) expression of TNF-α-, IL-6-, IL12p40- and IL-12p35 mRNAs in SiRNA-treated, LPS-stimulated BMDCs*. mRNAs were assayed by qRT-PCR in SiRNA-transfected BMDCs that were stimulated by LPS for various periods of time. The data presented correspond to 1 representative experiment out of 3 independent experiment.

Next, we asked whether JunB is involved in the transcriptional regulation of inflammatory cytokines such as TNF-α, IL-6 and IL-12 in LPS-stimulated BMDCs as their genes are well known targets of AP-1 in other cell types [23], [44], [45], [46], [47]. To this aim, we inhibited JunB induction upon siRNA transfection in BMDCs that were subsequently stimulated by LPS before kinetic analysis of mRNAs for TNF-α, IL-6 and IL-12 by qRT-PCR. Under our experimental conditions, the induction of JunB protein (**Figure 5A**) and *junb* mRNA (**Figure 5B**) was reduced by 70% by the anti-*junb* siRNA whereas a control siRNA had no effect (**Figure 5B**). This extent of repression was consistent with the fraction (70%) of transfected cells, as estimated from parallel transfection with a fluorescent control dsRNA (not shown). The data presented in **Figures 5C and 5D** show that, in the presence of the control siRNA, the mRNAs for TNF-α and IL-6 were induced rapidly and transiently with peaks of expression between 1 and 4 hours for TNF-α mRNA and by 4 hours for IL-6, i.e. after the onset of JunB protein induction (also see discussion). In contrast, in the presence of the anti-*junb* siRNA, inductions of these mRNAs were reduced by 50 to 80%, depending on the time point considered. IL-12 is made up of two chains: IL-12p40 and IL-12p35. Although the genes for the two chains are inducible by a variety of stimuli, only that for IL-12p40 is known to be AP-1-responsive. Interestingly, the induction of IL-12p40 mRNA (**Figure 5E**), but not that of IL-12p35 (**Figure 5F**), was inhibited by the *junb* siRNA. This was consistent with an involvement of JunB in the induction of one chain but not of the other, the latter serving as a negative control in the above experiments.

**Figure 6:**
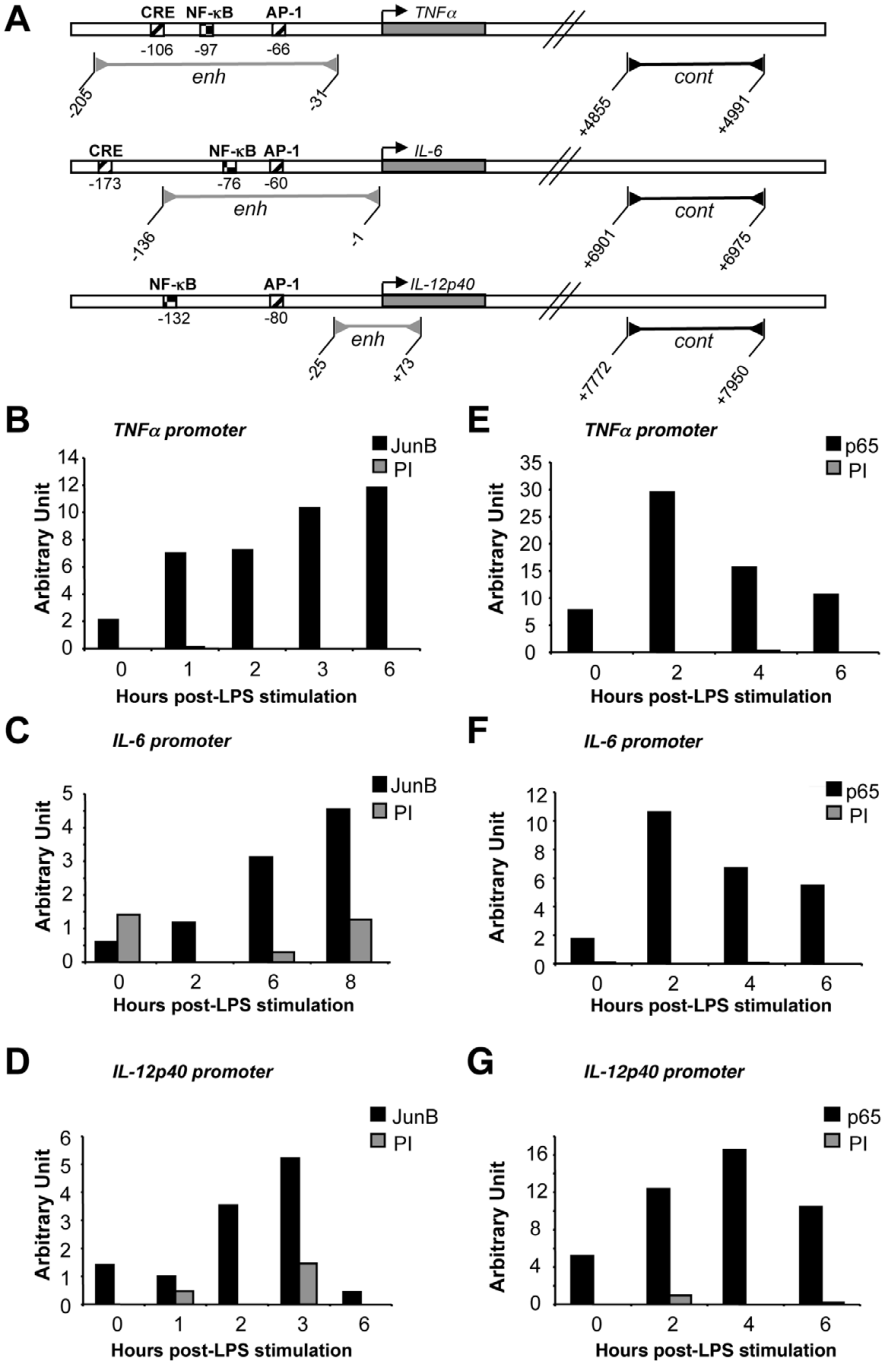
Binding of JunB and NF-κB/p65 to the promoters of TNFα, IL-6 and IL-12p40 genes in LPS-stimulated BMDCs. (A) *Binding sites for AP-1 and NF-κB in the promoter regions of TNFα, IL-6 and IL-12p40*. The grey bars with the inverted arrows (enh) indicate the enhancer-containing fragment which is amplified in ChIP experiments to visualize JunB and NF-κB binding. The black bars with the inverted arrows (cont) located downstream of the various genes indicate the amplified negative control fragments used in the ChIP experiments to exclude non-specific JunB and NF-κB binding. (B, C and D) *Binding of JunB to the promoter regions of the TNFα, IL-6 and IL-12p40 genes*. BMDCs were LPS-stimulated for various periods of time and ChIP experiments were carried out for assessing the presence of JunB in the cytokine gene promoter regions. PI corresponds to negative control immunoprecipitations with preimmune sera. Non-specific binding of JunB was excluded by qPCR analysis of a DNA fragment devoid of any AP-1 site and located downstream of each gene (not shown). (E, F and G) *Binding of NF-κB/p65 to promoter regions of the TNFα, IL-6 and IL-12p40 genes*. The experiments were carried as in B, C and D, except that an anti-NF-κB/p65 antiserum was used instead of the anti-JunB one. JunB and NF-κB bindings in the enhancer region are presented in arbitrary units as well as the parallel negative control ChIPs carried out with a preimmune serum (PI). Calculations were made with respect to the amplification of the “cont” negative control fragment for each gene.

AP-1/CRE responsive elements have already been identified in the promoter regions of the TNF-α, IL-6 and IL-12p40 genes ([23], [44], [45], [46], [47]; **Figures 6A**). We therefore asked by ChIP whether JunB bound specifically to these enhancer regions during the course of cytokine mRNA induction. The data presented in **Figures 6B–D** show that this was indeed the case. Taken with the above-described experiments, this strongly suggested that transient induction of TNF-α, IL-6 and IL-12p40 genes was contributed by JunB. As NF-κB binding sites crucial for the induction of these genes [23], [44], [45], [46], [47] are located in the vicinity of the AP-1 sites, we also verified inducible binding of NF-κB/p65 in ChIP experiments. Consistent with transient induction of NF-κB in DCs [1], [34], binding peaked by 2 hours post-stimulation for the TNFα and IL-6 genes and by 4 hours for the IL-12p40 gene (**Figures 6E–G**).

## Discussion

AP-1 has a recognized role in the control of immunity. However, it has not yet been subjected to in-depth investigation in DCs. As a first step to this study, we have asked here whether its JunB component might have a role in the induction of pro-inflammatory cytokine genes in response to *E. coli* LPS stimulation. Due to the scarcity of established DC cell lines and the bias associated with their use, we have conducted our experiments in primary mouse bone marrow-derived dendritic cells.

Our data show that the *junb* gene is rapidly and transiently induced in response to TLR4 signaling upon LPS activation of BMDCs. This is consistent with observations by others in macrophage- [25] and pre-B cell [19] lines as well as with previous transcriptomic analyses in DCs (see [7]). We also showed that *junb* induction in BMDCs is dependent on NF-κB as demonstrated by its inhibition by a pharmacological inhibitor of IKK and the inducible binding of NF-κB/p65 to the main *junb* enhancer region [31], [48], [49] as assayed by ChIP. Strengthening this conclusion, we observed in an LPS-stimulated mouse DC cell line (DC2.4; [50]) that a *junb* reporter gene was no longer transcriptionally induced in transient luciferase transactivation assays when its NF-kB sites were mutated (data not shown) in addition to BAY11-7085-inhibitable inductions of both JunB protein and *junb* mRNA similar to those in BMDCs and transient LPS-inducible binding of NF-κB/p65 to the *junb* enhancer. In other cellular backgrounds, the activation of *junb* transcription has been reported not to be due to enhanced initiation of transcription but rather to the release of already transcriptionally engaged RNA polymerases that are blocked just downstream (less than 100 nucleotides) of the transcription initiation site [51]. It will be interesting to address this point in future experiments. Noteworthy, JunB protein induction is much longer lasting than that of *junb* mRNA in LPS-stimulated BMDCs, which is largely contributed by high protein stability in this cell lineage. This constrasts with other situations where JunB has been shown instable but fits with the notion of its regulatable turnover [37].

Our RNA interference experiments have demonstrated an important role for JunB in the induction of the TNF-α, IL-6 and IL-12 pro-inflammatory cytokine mRNAs in LPS-stimulated BMDCs. This observation is interesting for two reasons: (i) among the Jun proteins, investigations have essentially been restricted to c-Jun when studying the transcriptional activation of such genes [37] and (ii) JunB is often considered as a transcriptional repressor, or a poor activator, despite its demonstrated direct role in the induction of cytokines like IL-2, IL-4 [27], [28], [29] and VEGF [31], or of the cyclin A gene [37], [52] in various cell contexts. Our ChIP analyses showed LPS-inducible association of JunB with the AP-1 responsive site of the TNF-α [45], [46], IL-6 [23], [44] and IL-12p40 [47] promoters that parallels the activation of these genes, as assayed by qRT-PCR. Taken with the above-mentioned siRNA experiments, this strongly argues for direct transcriptional activation of TNF-α, IL-6 and IL-12p40 genes by JunB. Not constituting a proof but consistent with this idea, preliminary data indicate that BMDC stimulation *via* TLR7 entails delayed induction of JunB, which is associated with delayed proinflammatory cytokines production (data not shown).

At least three important issues relate to the transcriptional partners of JunB in the transcriptional induction of TNF-α, IL-6 and IL-12p40 genes in LPS-stimulated BMDCs. The first issue is whether JunB acts as a homodimer under this condition or whether it has to heterodimerize with another AP-1-constituting proteins. The latter possibility is all the more to be taken into consideration that JunB affinity for itself is relatively low and that it can heterodimerize with a variety of other b-Zip proteins [10]. Illustrating this possibility in another cell system, MafA cooperates with JunB in Th2 T cells for inducing the IL-4 gene [29]. In BMDCs, Fra-2 might represent a good candidate for several reasons: (i) among the 4 Fos proteins, which are the highest affinity partners of the Juns [10], it is the only one to be expressed in LPS-treated BMDCs (unpublished data), which is a finding in agreement with absence of c-Fos expression recently observed in a similar setting [22], (ii) Fra-2 is induced with a kinetics paralleling that of JunB and (iii) it is found associated with JunB in co-immunoprecipitation experiments (unpublished data). Further experiments will explore this point. The second issue concerns the collaboration of JunB with NF-κB/p65. It is interesting to note that both JunB and NF-κB/p65 are found contemporarily bound to the TNF-α, IL-6 and IL-12p40 gene promoters when those are turned on. As NF-κB is an inducer of, not only TNF-α, IL-6 and IL-12p40 genes [23], [44], [45], [46], [47], but also of *junb* in BMDCs (this work), this points to the existence of a regulatory loop where NF-κB induces one of its transcriptional partner for subsequent induction of pro-inflammatory cytokine genes that constitutes an essential step in the DC activation process. Such a regulatory loop has already been observed upon LPS activation of the 70Z/3 pre-B cell line for induction of the CCR7 chemokine receptor [19], suggesting that this mechanism may apply in various cell types for induction of different types of genes. Moreover, inhibition of IKK by BAY11-7085 3 hours post-LPS stimulation (which permits inhibition of NF-κB after JunB induction) led to reduction of TNF-α, IL-6 and IL-12p40 gene transcription as assayed by qRT-PCR (data not shown). The third issue relates to the fact that induction of TNF-α, IL-6 and IL-12p40 mRNAs are not synchronous, IL-6 mRNA level increasing later than those of the other two mRNAs (**Figure 5**). This points to the existence of factor(s) differentially affecting the timing of transcriptional activation of these three genes. It is possible that one such factor(s) may operate by delaying binding of JunB to the IL-6 promoter as suggested by our ChIP experiments (**Figure 6B–D**).

Finally, it is important to wonder about whether JunB is always an activator of the TNF-α, IL-6 and IL-12p40 genes in BMDCs. In fact, (i) ChIP experiments (**Figure 6**) indicate that JunB is still present on the promoters of these genes after they have been transcriptionally switched off, as deduced from the reduction in their mRNA levels (**Figure 5**) and (ii) higher cytokine gene expression is observed 24 hours post-LPS stimulation in the presence of the anti-*junb* siRNA (**Figure 5**). As already mentioned in the introduction, certain AP-1 proteins, including JunB, can oscillate between activator and repressor states, depending on their post-translational modifications [16], [17], [27]. It will therefore be interesting to investigate this possibility for JunB in BMDCs. Alternatively, it is also possible that progressive inhibition of NF-κB after its peak of activation also contributes to turn JunB from an transcriptional activator state into a repressor one at the level of proinflammatory cytokine genes.

In conclusion, DCs are essential components of adaptive immune responses and represent innovative therapeutical tools against infectious diseases, cancer and allergies. It is therefore essential to elucidate the molecular basis of their genetic reprogramming upon stimulation of various sorts towards a better understanding of immunity and improvement of DC-based immunotherapies. As AP-1 is known to play a pivotal role in regulation of the immune system and has hardly been studied in DCs, we have addressed here the role of its JunB constituent in LPS-stimulated BMDCs. Our data indicate that JunB is crucial for the transcriptional induction of genes encoding TNF-α, IL-6 and IL-12p40 in LPS-stimulated BMDCs and acts in concert with NF-κB, which is not only its transcriptional partner, but also its necessary inducer in response to LPS-activated TLR4 signaling.
